# Ancestral Reconstruction

**DOI:** 10.1371/journal.pcbi.1004763

**Published:** 2016-07-12

**Authors:** Jeffrey B. Joy, Richard H. Liang, Rosemary M. McCloskey, T. Nguyen, Art F. Y. Poon

**Affiliations:** 1 BC Centre for Excellence in HIV/AIDS, Vancouver, British Columbia, Canada; 2 University of British Columbia, Department of Medicine, Vancouver, British Columbia, Canada

## Introduction

Ancestral reconstruction is the extrapolation back in time from measured characteristics of individuals (or populations) to their common ancestors. It is an important application of phylogenetics, the reconstruction and study of the evolutionary relationships among individuals, populations, or species to their ancestors. In the context of biology, ancestral reconstruction can be used to recover different kinds of ancestral character states, including the genetic sequence (ancestral sequence reconstruction), the amino acid sequence of a protein, the composition of a genome (e.g., gene order), a measurable characteristic of an organism (phenotype), and the geographic range of an ancestral population or species (ancestral range reconstruction). Nonbiological applications include the reconstruction of the vocabulary or phonemes of ancient languages [1] and cultural characteristics of ancient societies such as oral traditions [2] or marriage practices [3].

Ancestral reconstruction relies on a sufficiently realistic model of evolution to accurately recover ancestral states. No matter how well the model approximates the actual evolutionary history, however, one's ability to accurately reconstruct an ancestor deteriorates with increasing evolutionary time between that ancestor and its observed descendants. Additionally, more realistic models of evolution are inevitably more complex and difficult to calculate. Progress in the field of ancestral reconstruction has relied heavily on the exponential growth of computing power and the concomitant development of efficient computational algorithms (e.g., a dynamic programming algorithm for the joint maximum likelihood [ML] reconstruction of ancestral sequences [4]). Methods of ancestral reconstruction are often applied to a given phylogenetic tree that has already been inferred from the same data. While convenient, this approach has the disadvantage that its results are contingent on the accuracy of a single phylogenetic tree. In contrast, some researchers advocate a more computationally intensive Bayesian approach that accounts for uncertainty in tree reconstruction by evaluating ancestral reconstructions over many trees [5].

## History

The concept of ancestral reconstruction is often credited to Emile Zuckerkandl and Linus Pauling. Motivated by the development of techniques for determining the primary (amino acid) sequence of proteins by Frederick Sanger in 1955 [6], Pauling and Zuckerkandl postulated [7] that such sequences could be used to infer not only the phylogeny relating the observed protein sequences but also the ancestral protein sequence at the earliest point (root) of this tree. However, the idea of reconstructing ancestors from measurable biological characteristics had already been developing in the field of cladistics, one of the precursors of modern phylogenetics. Cladistic methods, which appeared as early as 1901, infer the evolutionary relationships of species on the basis of the distribution of shared characteristics, of which some are inferred to be descended from common ancestors. Furthermore, Theodoseus Dobzhansky and Alfred Sturtevant articulated the principles of ancestral reconstruction in a phylogenetic context in 1938, when inferring the evolutionary history of chromosomal inversions in *Drosophila pseudoobscura* [8]. Thus, ancestral reconstruction has its roots in several disciplines. Today, computational methods for ancestral reconstruction continue to be extended and applied in a diversity of settings so that ancestral states are being inferred not only for biological characteristics and the molecular sequences but also for the structure of folded proteins [9,10], the geographic location of populations and species (phylogeography) [11,12], and the higher-order structure of genomes [13].

## Methods and Algorithms

Any attempt at ancestral reconstruction begins with a phylogeny. In general, a phylogeny is a tree-based hypothesis about the order in which populations (referred to as taxa) are related by descent from common ancestors. Observed taxa are represented by the tips or terminal nodes of the tree that are progressively connected by branches to their common ancestors, which are represented by the branching points of the tree that are usually referred to as the ancestral or internal nodes. Eventually, all lineages converge to the most recent common ancestor of the entire sample of taxa. In the context of ancestral reconstruction, a phylogeny is often treated as though it were a known quantity (with Bayesian approaches being an important exception). Because there can be an enormous number of phylogenies that are nearly equally effective at explaining the data, reducing the subset of phylogenies supported by the data to a single representative, or point estimate, can be a convenient and sometimes necessary simplifying assumption. Ancestral reconstruction can be thought of as the direct result of applying a hypothetical model of evolution to a given phylogeny. When the model contains one or more free parameters, the overall objective is to estimate these parameters on the basis of measured characteristics among the observed taxa (sequences) that descended from common ancestors. Parsimony is an important exception to this paradigm: though it has been shown that there are mathematical models for which it is the ML estimator [14], at its core, it is simply based on the heuristic that changes in character state are rare, without attempting to quantify that rarity.

## Maximum Parsimony

**Fig 1:**
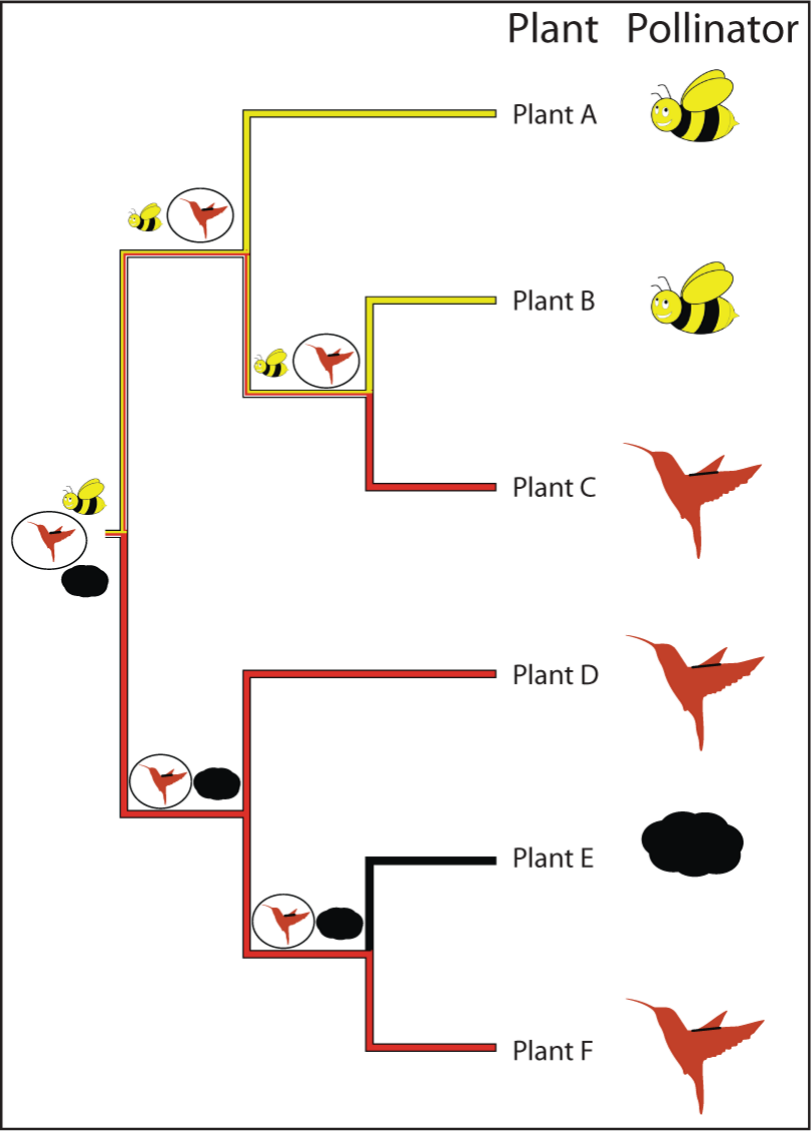
Phylogeny of a hypothetical genus of plants with pollination states of either “bees”, “hummingbirds”, or “wind” denoted by pictues at the tips. Pollination state nodes in the phylogenetic tree inferred under maximum parsimony are coloured on the branches leading into them (yellow represents “bee” pollination, red representing “hummingbird” pollination, and black representing “wind” pollination, dual coloured branches are equally parsimonious for the two states coloured). Assignment of “hummingbird” as the root state (because of prior knowledge from the fossil record) leads to the pattern of ancestral states represented by symbols at the nodes of the phylogeny, the state requiring the fewest number of changes to give rise to the pattern observed at the tips is circled at each node.

Parsimony, known colloquially as "Occam's razor," refers to the principle of selecting the simplest of competing hypotheses. In the context of ancestral reconstruction, parsimony endeavors to find the distribution of ancestral states within a given tree that minimizes the total number of character state changes that would be necessary to explain the states observed at the tips of the tree. This method of maximum parsimony) [15] is one of the earliest formalized algorithms for reconstructing ancestral states. Maximum parsimony can be implemented by one of several algorithms. One of the earliest examples is Fitch's method [16], which assigns ancestral character states by parsimony via two traversals of a rooted binary tree. The first stage is a postorder traversal that proceeds from the tips toward the root of a tree by visiting descendant (child) nodes before their parents. Initially, we are determining the set of possible character states *S*_*i*_ for the *i*-th ancestor based on the observed character states of its descendants. Each assignment is the set intersection) of the character states of the ancestor's descendants; if the intersection is the empty set, then it is the set union). In the latter case, it is implied that a character state change has occurred between the ancestor and one of its two immediate descendants. Each such event counts towards the algorithm's cost function, which may be used to discriminate among alternative trees on the basis of maximum parsimony. Next, a preorder traversal of the tree is performed, proceeding from the root towards the tips. Character states are then assigned to each descendant based on which character states it shares with its parent. Since the root has no parent node, one may be required to select a character state arbitrarily, specifically when more than one possible state has been reconstructed at the root. For example, consider a phylogeny recovered for a genus of plants containing six species, A–F (Fig 1), where each plant is pollinated by either a "bee," "hummingbird," or "wind." One obvious question is what the pollinators at deeper nodes were in the phylogeny of this genus of plants. Under maximum parsimony, an ancestral state reconstruction for this clade reveals that "hummingbird" is the most parsimonious ancestral state for the lower clade (plants D, E, F), that the ancestral states for the nodes in the top clade (plants A, B, C) are equivocal, and that both "hummingbird" or "bee" pollinators are equally plausible for the pollination state at the root of the phylogeny, supposing we have strong evidence from the fossil record that the root state is "hummingbird." Resolution of the root to "hummingbird" would yield the pattern of ancestral state reconstruction depicted by the symbols at the nodes (Fig 1) with the state requiring the fewest number of changes circled. Parsimony methods are intuitively appealing and highly efficient, such that they are still used in some cases to seed ML optimization algorithms with an initial phylogeny [17]. However, they suffer from several issues:

1. Variation in rates of evolution. Fitch's method assumes that changes between all character states are equally likely to occur; thus, any change incurs the same cost for a given tree. This assumption is often unrealistic and can limit the accuracy of such methods. For example, transitions) tend to occur more often than transversions in the evolution of nucleic acids. This assumption can be relaxed by assigning differential costs to specific character state changes, resulting in a weighted parsimony algorithm [18].
2. Rapid evolution. The upshot of the "minimum evolution" heuristic underlying such methods is that such methods assume that changes are rare and thus are inappropriate in cases where change is the norm rather than the exception [19,20].
3. Variation in time among lineages. Parsimony methods implicitly assume that the same amount of evolutionary time has passed along every branch of the tree. Thus, they do not account for variation in branch lengths in the tree, which are often used to quantify the passage of evolutionary or chronological time. This limitation makes the technique liable to infer that one change occurred on a very short branch rather than multiple changes occurring on a very long branch, for example [21]. This shortcoming is addressed by model-based methods (both ML and Bayesian methods) that infer the stochastic process of evolution as it unfolds along each branch of a tree [22].
4. Statistical justification. Without a statistical model underlying the method, its estimates do not have well-defined uncertainties [19,21,23].

## ML

ML methods of ancestral state reconstruction treat the character states at internal nodes of the tree as parameters and attempt to find the parameter values that maximize the probability of the data (the observed character states) given the hypothesis (a model of evolution and a phylogeny relating the observed sequences or taxa). Some of the earliest ML approaches to ancestral reconstruction were developed in the context of genetic sequence evolution [24,25]; similar models were also developed for the analogous case of discrete character evolution [26].

These approaches employ the same probabilistic framework as used to infer the phylogenetic tree [27]. In brief, the evolution of a genetic sequence is modelled by a time-reversible continuous time Markov process. In the simplest of these, all characters undergo independent state transitions (such as nucleotide substitutions) at a constant rate over time. This basic model is frequently extended to allow different rates on each branch of the tree. In reality, mutation rates may also vary over time (due, for example, to environmental changes); this can be modelled by allowing the rate parameters to evolve along the tree, at the expense of having an increased number of parameters. A model defines transition probabilities from states *i* to *j* along a branch of length *t* (in units of evolutionary time). The likelihood of a phylogeny is computed from a nested sum of transition probabilities that corresponds to the hierarchical structure of the proposed tree. At each node, the likelihood of its descendants is summed over all possible ancestral character states at that node:

$$L_{x}=\sum_{S_{x}\in \Omega }P(S_{x})\left(\sum_{S_{y}\in \Omega }P(S_{y}|S_{x},t_{xy})\;L_{y}\;\sum_{S_{z}\in \Omega }P(S_{z}|S_{x},t_{xz})\;L_{z}\right)$$

where we are computing the likelihood of the subtree rooted at node *x* with direct descendants *y* and *z*, *S*_*i*_ denotes the character state of the *i*-th node, *t*_*ij*_ is the branch length (evolutionary time) between nodes *i* and *j*, and *Ω* is the set of all possible character states (for example, the nucleotides A, C, G, and T). Thus, the objective of ancestral reconstruction is to find the assignment to *S*_*x*_ for all *x* internal nodes that maximizes the likelihood of the observed data for a given tree.

### Marginal and joint likelihood

Rather than compute the overall likelihood for alternative trees, the problem for ancestral reconstruction is to find the combination of character states at each ancestral node with the highest marginal ML. Generally speaking, there are two approaches to this problem. First, one may work upwards from the descendants of a tree to progressively assign the most likely character state to each ancestor taking into consideration only its immediate descendants. This approach is referred to as marginal reconstruction. It is akin to a greedy algorithm that makes the locally optimal choice at each stage of the optimization problem. While it can be highly efficient, it is not guaranteed to attain a globally optimal solution to the problem. Second, one may instead attempt to find the joint combination of ancestral character states throughout the tree that jointly maximizes the likelihood of the data. Thus, this approach is referred to as joint reconstruction. While it is not as rapid as marginal reconstruction, it is also less likely to be caught in the local optima in nonconvex objective functions that modern optimization methods and heuristics are designed to avoid. In the context of ancestral reconstruction, this means that a marginal reconstruction may assign a character state to the immediate ancestor that is locally optimal but deflects the joint distribution of ancestral character states away from the global optimum (for examples, see Pupko and colleagues [4]). Not surprisingly, joint reconstruction is more computationally complex than marginal reconstruction. Nevertheless, efficient algorithms for joint reconstruction have been developed with a time complexity that is generally linear with the number of observed taxa or sequences.

ML-based methods of ancestral reconstruction tend to provide greater accuracy than MP methods in the presence of variation in rates of evolution among characters (or across sites in a genome) [28,29]. However, these methods are not yet able to accommodate variation in rates of evolution over time, otherwise known as heterotachy. If the rate of evolution for a specific character accelerates on a branch of the phylogeny, then the amount of evolution that has occurred on that branch will be underestimated for a given length of the branch and assuming a constant rate of evolution for that character. In addition to that, it is difficult to distinguish heterotachy from variation among characters in rates of evolution [30].

Since ML (unlike maximum parsimony) requires the investigator to specify a model of evolution, its accuracy may be affected by the use of a grossly incorrect model (model misspecification). Furthermore, ML can only provide a single reconstruction of character states (what is often referred to as a "point estimate")—when the likelihood surface is highly nonconvex, comprising multiple peaks (local optima), then a single point estimate cannot provide an adequate representation, and a Bayesian approach may be more suitable.

## Bayesian Inference

Bayesian inference uses the likelihood of observed data to update the investigator's belief, or prior distribution, to yield the posterior distribution. In the context of ancestral reconstruction, the objective is to infer the posterior probabilities of ancestral character states at each internal node of a given tree. Moreover, one can integrate these probabilities over the posterior distributions over the parameters of the evolutionary model and the space of all possible trees. This can be expressed as an application of Bayes' theorem:

$$\begin{array}{l} P(S|D,\theta )=\frac{P(D|S,\theta )\;P(S|\theta )}{P(D|\theta )}\\ \;\propto P(D|S,\theta )\;P(S|\theta )\;P(\theta ), \end{array}$$

where *S* represents the ancestral states, *D* corresponds to the observed data, and *θ* represents both the evolutionary model and the phylogenetic tree. *P*(*D*|*S*,*θ*) is the likelihood of the observed data that can be computed by Felsenstein's pruning algorithm as given above. *P*(*S*|*θ*) is the prior probability of the ancestral states for a given model and tree. Finally, *P*(*D*|*θ*) is the probability of the data for a given model and tree, integrated over all possible ancestral states. We have given two formulations to emphasize the two different applications of Bayes' theorem, which we discuss in the following section.

### Empirical and hierarchical Bayes

One of the first implementations of a Bayesian approach to ancestral sequence reconstruction was developed by Yang and colleagues, where the ML estimates of the evolutionary model and tree, respectively, were used to define the prior distributions. Thus, their approach is an example of an empirical Bayes method to compute the posterior probabilities of ancestral character states; this method was first implemented in the software package PAML [31]. In terms of the above Bayesian rule formulation, the empirical Bayes method fixes to the empirical estimates of the model and tree obtained from the data, effectively dropping from the posterior likelihood and prior terms of the formula. Moreover, Yang and colleagues [24] used the empirical distribution of site patterns (i.e., assignments of nucleotides to tips of the tree) in their alignment of observed nucleotide sequences in the denominator in place of exhaustively computing *P*(*D*) over all possible values of *S*, given *θ*. Computationally, the empirical Bayes method is akin to the ML reconstruction of ancestral states except that, rather than searching for the ML assignment of states based on their respective probability distributions at each internal node, the probability distributions themselves are reported directly.

Empirical Bayes methods for ancestral reconstruction require the investigator to assume that the evolutionary model parameters and tree are known without error. When the size or complexity of the data makes this an unrealistic assumption, it may be more prudent to adopt the fully hierarchical Bayesian approach and infer the joint posterior distribution over the ancestral character states, model, and tree [32]. Huelsenbeck and Bollback [32] first proposed a hierarchical Bayes method to ancestral reconstruction by using Markov chain Monte Carlo (MCMC) methods to sample ancestral sequences from this joint posterior distribution. A similar approach was also used to reconstruct the evolution of symbiosis with algae in fungal species (lichenization) [33]. For example, the Metropolis-Hastings algorithm for MCMC explores the joint posterior distribution by accepting or rejecting parameter assignments on the basis of the ratio of posterior probabilities.

Put simply, the empirical Bayes approach calculates the probabilities of various ancestral states for a specific tree and model of evolution. By expressing the reconstruction of ancestral states as a set of probabilities, one can directly quantify the uncertainty for assigning any particular state to an ancestor. On the other hand, the hierarchical Bayes approach averages these probabilities over all possible trees and models of evolution, in proportion to how likely these trees and models are, given the data that has been observed.

However, whether the hierarchical Bayes method confers a substantial advantage in practice remains controversial [34]. Moreover, this fully Bayesian approach is limited to analyzing relatively small numbers of sequences or taxa because the space of all possible trees rapidly becomes too vast, making it computationally infeasible for chain samples to converge in a reasonable amount of time.

## Calibration

Ancestral reconstruction can be informed by the observed states in historical samples of known age, such as fossils or archival specimens. Since the accuracy of ancestral reconstruction generally decays with increasing time, the use of such specimens provides data that are closer to the ancestors being reconstructed and will most likely improve the analysis, especially when rates of character change vary through time. This concept has been validated by an experimental evolutionary study in which replicate populations of bacteriophage T7 were propagated to generate an artificial phylogeny [35]. In revisiting these experimental data, Oakley and Cunningham [36] found that maximum parsimony methods were unable to accurately reconstruct the known ancestral state of a continuous character (plaque size); these results were verified by computer simulation. This failure of ancestral reconstruction was attributed to a directional bias in the evolution of plaque size (from large to small plaque diameters) that required the inclusion of "fossilized" samples to address.

Studies of both mammalian carnivores [37] and fishes [38] have demonstrated that, without incorporating fossil data, the reconstructed estimates of ancestral body sizes are unrealistically large. Moreover, Graham Slater and colleagues showed using *Caniform carnivorans* that incorporating fossil data into prior distributions improved both the Bayesian inference of ancestral states and evolutionary model selection, relative to analyses using only contemporaneous data [39].

## Models

Many models have been developed to estimate ancestral states of discrete and continuous characters from extant descendants [40]. Such models assume that the evolution of a trait through time may be modelled as a stochastic process. For discrete-valued traits (such as "pollinator type"), this process is typically taken to be a Markov chain; for continuous-valued traits (such as "brain size"), the process is frequently taken to be a Brownian motion or an Ornstein–Uhlenbeck process. Using this model as the basis for statistical inference, one can now use ML methods or Bayesian inference to estimate the ancestral states.

### Discrete-state models

**Fig 2:**
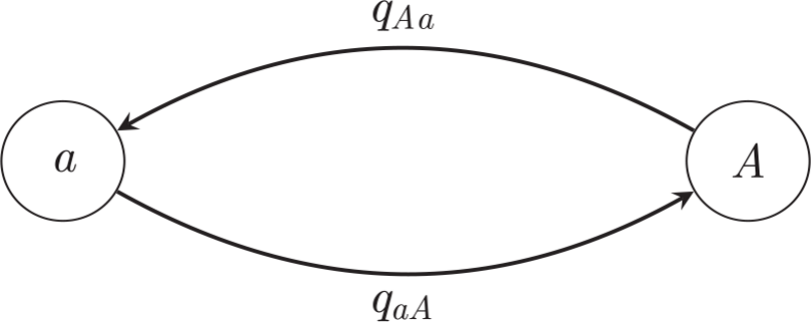
**A general two-state Markov chain representing the rate of jumps from allele *a* to allele *A*.** The different types of jumps are allowed to have different rates.

Suppose the trait in question may fall into one of *k* states, labeled 1,…,*k*. The typical means of modelling evolution of this trait is via a continuous-time Markov chain, which may be briefly described as follows (cf. Fig 2). Each state has associated to it rates of transition to all of the other states. The trait is modelled as stepping between the *k* states; when it reaches a given state, it starts an exponential "clock" for each of the other states that it can step to. It then "races" the clocks against each other, and it takes a step towards the state whose clock is the first to ring. In such a model, the parameters are the transition rates ***q*** = {*q*_*ij*_: 1 ≤ *i*,*j* ≤ *k*, *i* ≠ *j*}, which can be estimated using, for example, ML methods, where one maximizes over the set of all possible configurations of states of the ancestral nodes.

In order to recover the state of a given ancestral node in the phylogeny (call this node *α*) by ML, the procedure is: find the ML estimate $\hat{q}$ of ***q***; then compute the likelihood of each possible state for *α* conditioning on $q=\hat{q}$; finally, choose the ancestral state that maximizes this [19]. One may also use this substitution model as the basis for a Bayesian inference procedure, which would consider the posterior belief in the state of an ancestral node given some user-chosen prior.

**Fig 3:**
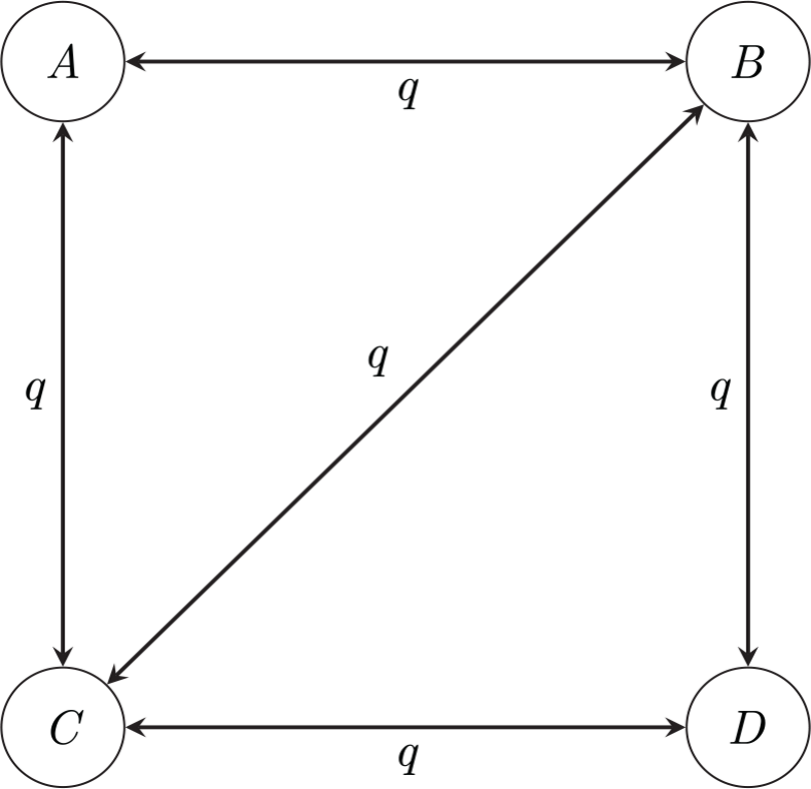
Example of a four-state 1 parameter Markov chain model. Note that in this diagram, transitions between states *A* and *D* have been disallowed; it is conventional to not draw the arrow rather than to draw it with a rate of 0.

**Fig 4:**
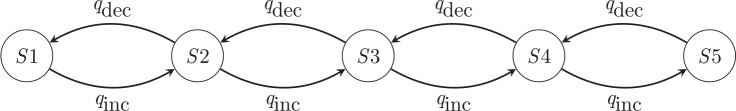
Graphical representation of an asymmetrical five-state 2-parameter Markov chain model.

Because such models may have as many as *k*(*k* − 1) parameters, overfitting may be an issue. Some common choices that reduce the parameter space are:

- Markov *k*-state 1 parameter model (Fig 3): this model is the reverse-in-time *k*-state counterpart of the Jukes–Cantor model. In this model, all transitions have the same rate *q*, regardless of their start and end states. Some transitions may be disallowed by declaring that their rates are simply 0; this may be the case, for example, if certain states cannot be reached from other states in a single transition.

- Asymmetrical Markov *k*-state 2 parameter model (Fig 4): in this model, the state space is ordered (so that, for example, state 1 is smaller than state 2, which is smaller than state 3, and transitions may only occur between adjacent states). This model contains two parameters *q*_inc_ and *q*_dec_: one for the rate of increase of state (e.g., 0 to 1, 1 to 2, etc.) and one for the rate of decrease in state (e.g., from 2 to 1, 1 to 0, etc.).

### Example: Binary state speciation and extinction model

The binary state speciation and extinction model [41] (BiSSE) is a discrete-space model that does not directly follow the framework of those mentioned above. It allows estimation of ancestral binary character states jointly with diversification rates associated with different character states; it may also be straightforwardly extended to a more general multiple-discrete-state model. In its most basic form, this model involves six parameters: two speciation rates (one each for lineages in states 0 and 1); similarly, two extinction rates; and two rates of character change. This model allows for hypothesis testing on the rates of speciation/extinction/character change, at the cost of increasing the number of parameters.

### Continuous-state models

In the case where the trait instead takes nondiscrete values, one must instead turn to a model where the trait evolves as some continuous process. Inference of ancestral states by ML (or by Bayesian methods) would proceed as above but with the likelihoods of transitions in state between adjacent nodes given by some other continuous probability distribution.

**Fig 5:**
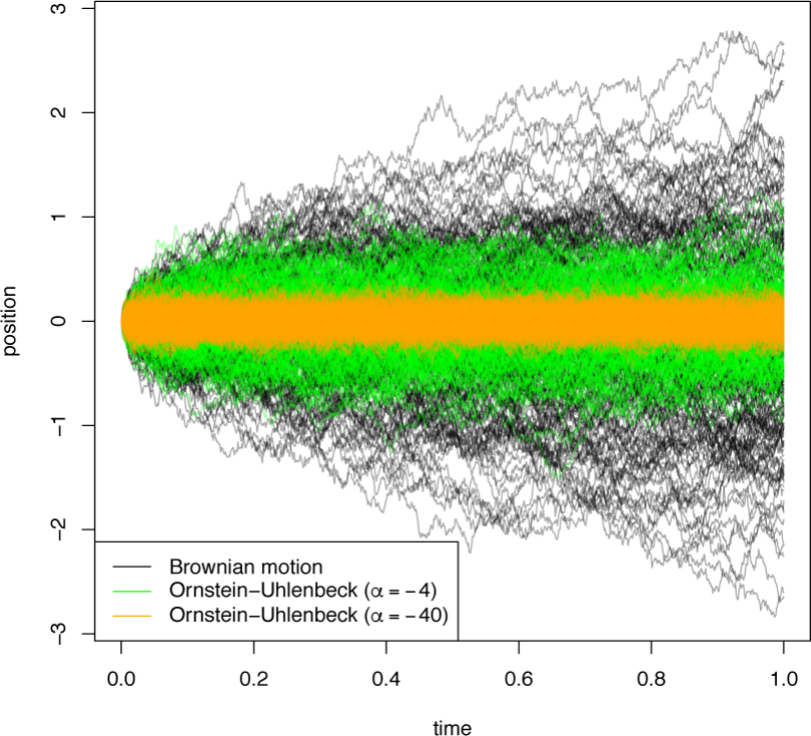
**Plots of 200 trajectories of each of: Brownian motion with drift 0 and *σ*^2^ = 1 (black); Ornstein–Uhlenbeck with *σ*^2^ = 1 and *α* = −4 (green); and Ornstein–Uhlenbeck with *σ*^2^ = 1 and *α* = −40 (orange)**.

- Brownian motion: in this case, if nodes *U* and *V* are adjacent in the phylogeny (say *U* is the ancestor of *V*) and separated by a branch of length *t*, the likelihood of a transition from *U* being in state *x* to *V* being in state *y* is given by a Gaussian density with mean 0 and variance *σ*^2^*t*. In this case, there is only one parameter (*σ*^2^), and the model assumes that the trait evolves freely without a bias toward increase or decrease, and that the rate of change is constant throughout the branches of the phylogenetic tree [42].
- Ornstein–Uhlenbeck process: in brief, an Ornstein–Uhlenbeck process is a continuous stochastic process that behaves like a Brownian motion, but attracted toward some central value, where the strength of the attraction increases with the distance from that value. This is useful for modelling scenarios where the trait is subject to stabilizing selection around a certain value (say 0). Under this model, the above-described transition of *U* being in state *x* to *V* being in state *y* would have a likelihood defined by the transition density of an Ornstein–Uhlenbeck process with two parameters: *σ*^2^, which describes the variance of the driving Brownian motion, and *α*, which describes the strength of its attraction to 0. As *α* tends to 0, the process is less and less constrained by its attraction to 0 and the process becomes a Brownian motion (Fig 5). Because of this, the models may be nested, and log-likelihood ratio tests discerning which of the two models is appropriate may be carried out.

- Stable models of continuous character evolution [43]: though Brownian motion is appealing and tractable as a model of continuous evolution, it does not permit non-neutrality in its basic form, nor does it provide for any variation in the rate of evolution over time. Instead, one may use a stable process, one whose values at fixed times are distributed as stable distributions, to model the evolution of traits. Stable processes, roughly speaking, behave as Brownian motions that also incorporate discontinuous jumps. This allows us to appropriately model scenarios in which short bursts of fast trait evolution are expected. In this setting, ML methods are poorly suited because of a rugged likelihood surface and because the likelihood may be made arbitrarily large, so Bayesian methods are more appropriate.

## Applications

### Character evolution

#### Behaviour and life history evolution

*Diet reconstruction in Galapagos finches*: Both phylogenetic and character data are available for the radiation of finches inhabiting the Galapagos Islands. These data allow testing of hypotheses concerning the timing and ordering of character state changes through time via ancestral state reconstruction. During the dry season, the diets of the 13 species of Galapagos finches may be assorted into three broad diet categories, first those that consume grain-like foods are considered “granivores,” those that ingest arthropods are termed “insectivores,” and those that consume vegetation are classified as “folivores” [19]. Dietary ancestral state reconstruction under maximum parsimony recovers two major shifts from an insectivorous state: one to granivory and one to folivory. Maximum-likelihood ancestral state reconstruction recovers broadly similar results, with one significant difference: the common ancestor of the tree finch (*Camarhynchus*) and ground finch (*Geospiza*) clades is most likely granivorous rather than insectivorous (as judged by parsimony). In this case, this difference between ancestral states returned by maximum parsimony and ML likely occurs as a result of the fact that ML estimates consider branch lengths of the phylogenetic tree [19].

#### Morphological character evolution mammalian body mass

In an analysis of the body mass of 1,679 placental mammal species comparing stable models of continuous character evolution to Brownian motion models, Elliot and Mooers [43] showed that the evolutionary process describing mammalian body mass evolution is best characterized by a stable model of continuous character evolution, which accommodates rare changes of large magnitude. Under a stable model, ancestral mammals retained a low body mass through early diversification, with large increases in body mass coincident with the origin of several orders of large body massed species (e.g., ungulates). By contrast, simulation under a Brownian motion model recovered a less realistic, order of magnitude larger body mass among ancestral mammals, requiring significant reductions in body size prior to the evolution of orders exhibiting small body size (e.g., Rodentia). Thus, stable models recover a more realistic picture of mammalian body mass evolution by permitting large transformations to occur on a small subset of branches [43].

#### Correlated character evolution

Comparative methods (inferences drawn through comparison of related taxa) are often used to identify biological characteristics that do not evolve independently, which can reveal an underlying dependence. For example, the evolution of the shape of a finch's beak may be associated with its foraging behaviour. However, it is not advisable to search for these associations by the direct comparison of measurements or genetic sequences, as these observations are not independent because of their descent from common ancestors. For discrete characters, this problem was first addressed in the framework of maximum parsimony by evaluating whether two characters tended to undergo a change on the same branches of the tree [44,45]. Felsenstein identified this problem for continuous character evolution and proposed a solution similar to ancestral reconstruction, in which the phylogenetic structure of the data was accommodated by directing the analysis on "independent contrasts" between nodes of the tree related by nonoverlapping branches [23].

#### Molecular evolution

On a molecular level, amino acid residues at different locations of a protein may evolve nonindependently, because they have a direct physicochemical interaction, or indirectly by their interactions with a common substrate, or through long-range interactions in the protein structure. Conversely, the folded structure of a protein could potentially be inferred from the distribution of residue interactions [46]. One of the earliest applications of ancestral reconstruction, to predict the three-dimensional structure of a protein through residue contacts, was published by Shindyalov and colleagues [47]. Phylogenies relating 67 different protein families were generated by a distance-based clustering method (unweighted pair group method with arithmetic mean, UPGMA), and ancestral sequences were reconstructed by parsimony. The authors reported a weak but significant tendency for coevolving pairs of residues to be colocated in the known three-dimensional structure of the proteins.

More recently, this concept has been applied to identify coevolving residues in protein sequences using more advanced methods for the reconstruction of phylogenies and ancestral sequences. For example, ancestral reconstruction has been used to identify coevolving residues in proteins encoded by RNA virus genomes, particularly in the human immunodeficiency virus (HIV) [48–50].

#### Vaccine design

RNA viruses such as HIV evolve at an extremely rapid rate, orders of magnitude faster than mammals or birds. For these organisms, ancestral reconstruction can be applied on a much shorter time scale; for example, in order to reconstruct the global or regional progenitor of an epidemic that has spanned decades rather than millions of years. A team around Brian Gaschen [51] proposed that such reconstructed strains be used as targets for vaccine design efforts as opposed to sequences isolated from patients in the present day. Because HIV is extremely diverse, a vaccine designed to work on one patient's viral population might not work for a different patient, because the evolutionary distance between these two viruses may be large. However, their most recent common ancestor is closer to each of the two viruses than they are to each other. Thus, a vaccine designed for a common ancestor could have a better chance of being effective for a larger proportion of circulating strains. Another team took this idea further by developing a center-of-tree (COT) reconstruction method to produce a sequence whose total evolutionary distance to contemporary strains is as small as possible [52]. Strictly speaking, this method was not ancestral reconstruction, as the COT sequence does not necessarily represent a sequence that has ever existed in the evolutionary history of the virus. However, Rolland and colleagues did find that, in the case of HIV, the COT virus was functional when synthesized. Similar experiments with synthetic ancestral sequences obtained by ML reconstruction have likewise shown that these ancestors are both functional and immunogenic, lending some credibility to these methods [53,54]. Furthermore, ancestral reconstruction can potentially be used to infer the genetic sequence of the transmitted HIV variants that have gone on to establish the next infection, with the objective of identifying distinguishing characteristics of these variants (as a nonrandom selection of the transmitted population of viruses) that may be targeted for vaccine design [55].

#### Genome rearrangements

Rather than inferring the ancestral DNA sequence, one may be interested in the larger-scale molecular structure of an ancestral genome. This problem is often approached in a combinatorial framework, by modelling genomes as permutations of genes or homologous regions. Various operations are allowed on these permutations, such as an inversion (a segment of the permutation is reversed in-place), deletion) (a segment is removed), or transposition (a segment is removed from one part of the permutation and spliced in somewhere else). The "genome rearrangement problem," first posed by Watterson and colleagues [13], asks: given two genomes (permutations) and a set of allowable operations, what is the shortest sequence of operations that will transform one genome into the other? A generalization of this problem applicable to ancestral reconstruction is the "multiple genome rearrangement problem" [56]: given a set of genomes and a set of allowable operations, find (i) a binary tree with the given genomes as its leaves, and (ii) an assignment of genomes to the internal nodes of the tree, such that the total number of operations across the whole tree is minimized. This approach is similar to parsimony, except that the tree is inferred along with the ancestral sequences. Unfortunately, even the single genome rearrangement problem is NP-hard [57], although it has received much attention in mathematics and computer science (for a review, see Fertin and colleagues [58]).

#### Spatial applications migration

Ancestral reconstruction is not limited to biological traits. Spatial location is also a trait, and ancestral reconstruction methods can infer the locations of ancestors of the individuals under consideration. Such techniques were used by Lemey and colleagues to geographically trace the ancestors of 192 Avian influenza A-H5N1 strains sampled from 20 localities in Europe and Asia and of 101 rabies virus sequences sampled across 12 African countries [12].

Treating locations as discrete states (countries, cities, etc.) allows for the application of the discrete-state models described above. However, unlike in a model where the state space for the trait is small, there may be many locations, and transitions between certain pairs of states may rarely or never occur; for example, migration between distant locales may never happen directly if air travel between the two places does not exist, so such migrations must pass through intermediate locales first. This means that there could be many parameters in the model which are zero or close to zero. To this end, Lemey and colleagues used a Bayesian procedure to not only estimate the parameters and ancestral states but also to select which migration parameters are not zero; their work suggests that this procedure does lead to more efficient use of the data. They also explore the use of prior distributions that incorporate geographical structure or hypotheses about migration dynamics, finding that those they considered had little effect on the findings.

Using this analysis, the team around Lemey found that the most likely hub of diffusion of A-H5N1 is Guangdong, with Hong Kong also receiving posterior support. Further, their results support the hypothesis of long-standing presence of African rabies in West Africa [12].

#### Species ranges

Inferring historical biogeographic patterns often requires reconstructing ancestral ranges of species on phylogenetic trees [59]. For instance, a well-resolved phylogeny of plant species in the genus *Cyrtandra* [59] was used together with information of their geographic ranges to compare four methods of ancestral range reconstruction. The team compared Fitch parsimony [16] (FP; parsimony), stochastic mapping (SM; ML) [60], dispersal-vicariance analysis [61] (DIVA; parsimony), and dispersal-extinction-cladogenesis (DEC; maximum-likelihood) [11,62]. Results indicated that both parsimony methods performed poorly, which was likely due to the fact that parsimony methods do not consider branch lengths. Both maximum-likelihood methods performed better; however, DEC analyses that additionally allow incorporation of geological priors gave more realistic inferences about range evolution in *Cyrtandra* relative to other methods [59].

Another ML method recovers the phylogeographic history of a gene by reconstructing the ancestral locations of the sampled taxa [63]. This method assumes a spatially explicit random walk model of migration to reconstruct ancestral locations given the geographic coordinates of the individuals represented by the tips of the phylogenetic tree. When applied to a phylogenetic tree of chorus frogs *Pseudacris feriarum*, this method recovered recent northward expansion, higher per-generation dispersal distance in the recently colonized region, a noncentral ancestral location, and directional migration.

**Fig 6:**
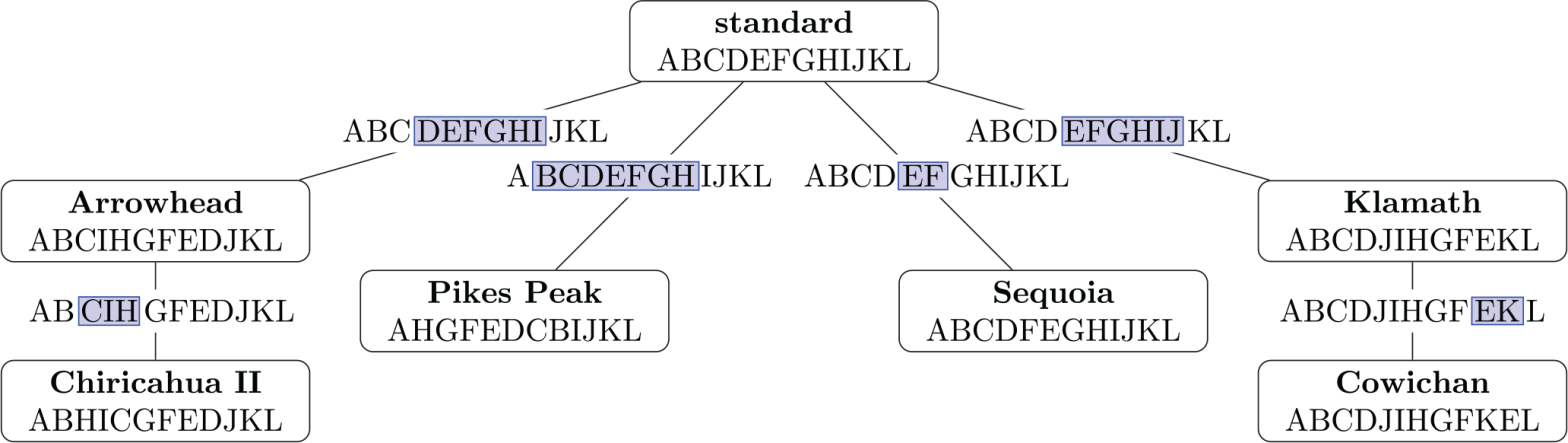
Phylogeny of seven regional strains of *Drosophila pseudoobscura*, as inferred by Sturtevant and Dobzhansky [64]. Displayed sequences do not correspond to the original paper, but were derived from the notation in the authors' companion paper [8] as follows: A (63A–65B), B (65C–68D), C (69A–70A), D (70B–70D), E (71A–71B), F (71A–73C), G (74A–74C), H (75A–75C), I (76A–76B), J (76C–77B), K (78A–79D), L (80A–81D). Inversions inferred by the authors are highlighted in blue along branches.

The first consideration of the multiple genome rearrangement problem, long before its formalization in terms of permutations, was presented by Sturtevant and Dobzhansky in 1936 (Fig 6) [64]. They examined genomes of several strains of fruit fly from different geographic locations, and observed that one configuration, which they called "standard," was the most common throughout all the studied areas. Remarkably, they also noticed that four different strains could be obtained from the standard sequence by a single inversion, and two others could be related by a second inversion. This allowed them to hypothesize a phylogeny for the sequences and to infer that the standard sequence was probably also the ancestral one.

#### Linguistic evolution

Reconstructions of the words and phenomes of ancient protolanguages such as Proto-Indo-European have been performed based on the observed analogues in present-day languages. Typically, these analyses are carried out manually using the "comparative method" [65]. First, words from different languages with a common etymology (cognates) are identified in the contemporary languages under study, analogous to the identification of orthologous biological sequences. Second, correspondences between individual sounds in the cognates are identified, a step similar to biological sequence alignment, although performed manually. Finally, likely ancestral sounds are hypothesized by manual inspection and various heuristics (such as the fact that most languages have both nasal and non-nasal vowels) [65].

### Software

There are many software packages available that perform ancestral state reconstruction. Generally, these software packages have been developed and maintained through the efforts of scientists in related fields and released under free software licenses. The following table is not meant to be a comprehensive itemization of all available packages, but provides a representative sample of the extensive variety of packages that implement methods of ancestral reconstruction with different strengths and features (Table 1).

**Table 1:** List of software for ancestral reconstruction.

Name	Methods	Platform	Supported Input Formats	Character Types	Continuous (C) or Discrete (D) Characters	Software Licence
PAML	ML	Unix, Mac, Win	PHYLIP, NEXUS, FASTA	Nucleotide, Protein	D	Proprietary
BEAST2	Bayesian	Unix, Mac, Win	NEXUS, BEAST XML	Nucleotide, Protein, Geographic	C, D	GNU Lesser General Public License
APE	ML	Unix, Mac, Win	NEXUS, FASTA, CLUSTAL	Nucleotide, Protein	C, D	GNU General Public License
Diversitree	ML	Unix, Mac, Win	NEXUS	Qualitative and quantitative traits, Geographic	C, D	GNU General Public License, version 2
HyPhy	ML	Unix, Mac, Win	MEGA, NEXUS, FASTA, PHYLIP	Nucleotide, Protein (customizable)	D	GNU Free Documentation License 1.3
BayesTraits	Bayesian	Unix, Mac, Win	TSV or space delimited table. Rows are species, columns are traits.	Qualitative and quantitative traits	C, D	Creative Commons Attribution License
Lagrange	ML	Linux, Mac, Win	TSV/CSV of species regions. Rows are species and columns are geographic regions	Geographic	-	GNU General Public License, version 2
Mesquite	Parsimony, ML	Unix, Mac, Win	Fasta, NBRF, Genbank, PHYLIP, CLUSTAL, TSV	Nucleotide, Protein,Geographic	C, D	Creative Commons Attribution 3.0 License
Phylomapper	ML, Bayesian (version 2)	Unix, Mac, Win	NEXUS	Geographic, Ecological niche	C, D	-
Ancestors	ML	Web	Fasta	Nucleotide (indels)	D	-
Phyrex	Maximum Parsimony	Linux	Fasta	Gene expression	C, D	Proprietary
SIMMAP	SM	Mac	XML-like format	Nucleotide, qualitative traits	D	Proprietary
MrBayes	Bayesian	Unix, Mac, Win	NEXUS	Nucleotide, Protein	D	GNU General Public License
PARANA	Maximum Parsimony	Unix, Mac, Win	Newick	Biological networks	D	Apache License
PHAST (PREQUEL)	ML	Unix, Mac, Win	Multiple Alignment	Nucleotide	D	BSD License
RASP	ML, Bayesian	Unix, Mac, Win	Newick	Geographic	D	-
VIP	Maximum Parsimony	Linux, Win	Newick	Geographic	D (grid)	GPL Creative Commons
FastML	ML	Web, Unix	Fasta	Nucleotide, Protein	D	Copyright
MLGO	ML	Web	Custom	Gene order permutation	D	GNU
BADGER	Bayesian	Unix, Mac, Win	Custom	Gene order permutation	D	GNU GPL version 2
COUNT	Maximum Parsimony	Unix, Mac, Win	Tab-delimited text file of rows for taxa and count data in columns	Count data (homolog family size)	D	BSD
MEGA	Maximum parsimony, ML	Mac, Win	MEGA	Nucleotide, Protein	D	Proprietary
ANGES	Local Parsimony	Unix	Custom	Genome maps	D	GNU General Public License, version 3
EREM	ML	Win, Unix, Matlab module	Custom text format for model parameters, tree, observed character values	Binary	D	None specified, although site indicates software is freely available

## Package Descriptions

### Molecular evolution

The majority of these software packages are designed for analyzing genetic sequence data. For example, PAML [31] is a collection of programs for the phylogenetic analysis of DNA and protein sequence alignments by ML. Ancestral reconstruction can be performed using the codeml program. HyPhy, Mesquite, and MEGA are also software packages for the phylogenetic analysis of sequence data, but are designed to be more modular and customizable. HyPhy [66] implements a joint ML method of ancestral sequence reconstruction [4] that can be readily adapted to reconstructing a more generalized range of discrete ancestral character states such as geographic locations by specifying a customized model in its batch language. Mesquite [67] provides ancestral state reconstruction methods for both discrete and continuous characters using both maximum parsimony and ML methods. It also provides several visualization tools for interpreting the results of ancestral reconstruction. MEGA [68] is a modular system, too, but places greater emphasis on ease-of-use than customization of analyses. As of version 5, MEGA allows the user to reconstruct ancestral states using maximum parsimony, ML, and empirical Bayes methods [68].

The Bayesian analysis of genetic sequences may confer greater robustness to model misspecification. MrBayes [69] allows inference of ancestral states at ancestral nodes using the full hierarchical Bayesian approach. The PREQUEL program distributed in the PHAST package performs comparative evolutionary genomics using ancestral sequence reconstruction [70]. SIMMAP stochastically maps mutations on phylogenies [71]. BayesTraits [26] analyses discrete or continuous characters in a Bayesian framework to evaluate models of evolution, reconstruct ancestral states, and detect correlated evolution between pairs of traits.

#### Other character types

Other software packages are more oriented towards the analysis of qualitative and quantitative traits (phenotypes). For example, the ape package [72] in the statistical computing environment R also provides methods for ancestral state reconstruction for both discrete and continuous characters through the *ace* function, including ML. Note that *ace* performs reconstruction by computing scaled conditional likelihoods instead of the marginal or joint likelihoods used by other ML-based methods for ancestral reconstruction, which may adversely affect the accuracy of reconstruction at nodes other than the root. Phyrex implements a maximum parsimony-based algorithm to reconstruct ancestral gene expression profiles in addition to a ML method for reconstructing ancestral genetic sequences (by wrapping around the baseml function in PAML) [73].

Several software packages also reconstruct phylogeography. BEAST (Bayesian Evolutionary Analysis by Sampling Trees [74]) provides tools for reconstructing ancestral geographic locations from observed sequences annotated with location data using Bayesian MCMC sampling methods. Diversitree [75] is an R package providing methods for ancestral state reconstruction under Mk2 (a continuous time Markov model of binary character evolution [76]) and BiSSE models. Lagrange performs analyses on reconstruction of geographic range evolution on phylogenetic trees [11]. Phylomapper [63] is a statistical framework for estimating historical patterns of gene flow and ancestral geographic locations. RASP [77] infers ancestral state using statistical DIVA, Lagrange, Bayes-Lagrange, BayArea, and BBM methods. VIP [78] infers historical biogeography by examining disjunct geographic distributions.

Genome rearrangements provide valuable information in comparative genomics between species. ANGES [79] compares extant-related genomes through ancestral reconstruction of genetic markers. BADGER [80] uses a Bayesian approach to examining the history of gene rearrangement. Count [81] reconstructs the evolution of the size of gene families. EREM [82] analyses the gain and loss of genetic features encoded by binary characters. PARANA [83] performs parsimony-based inference of ancestral biological networks that represent gene loss and duplication.

#### Web applications

Finally, there are several web server-based applications that allow investigators to use ML methods for ancestral reconstruction of different character types without having to install any software. For example, Ancestors [84] is a web server for ancestral genome reconstruction by the identification and arrangement of syntenic regions. FastML [85] is a web server for probabilistic reconstruction of ancestral sequences by ML that uses a gap character model for reconstructing indel variation. MLGO [86] is a web server for ML gene order analysis.

## Future Directions

The development and application of computational algorithms for ancestral reconstruction continues to be an active area of research across disciplines. For example, the reconstruction of sequence insertions and deletions (indels) has lagged behind the more straightforward application of substitution models. Bouchard-Côté and Jordan recently described a new model (the Poisson Indel Process [87]) that represents an important advance on the archetypal Thorne-Kishino-Felsenstein model of indel evolution [88]. In addition, the field is being driven forward by rapid advances in the area of next-generation sequencing technology, where sequences are generated from millions of nucleic acid templates by extensive parallelization of sequencing reactions in a custom apparatus. These advances have made it possible to generate a "deep" snapshot of the genetic composition of a rapidly-evolving population, such as RNA viruses [89] or tumour cells [90], in a relatively short amount of time. At the same time, the massive amount of data and platform-specific sequencing error profiles has created new bioinformatic challenges for processing these data for ancestral sequence reconstruction.

The version history of the text file and the peer reviews (and response to reviews) are available as supporting information in S1 and S2 Texts.

## Supporting Information

S1 Text Version history of the text file. (XML)

S2 Text Peer reviews and response to reviews. Human-readable versions of the reviews and authors' responses are available as comments on this article.
(XML)

## Abbreviations

BEAST: Bayesian Evolutionary Analysis by Sampling Trees
BiSSE: binary state speciation and extinction model
COT: center-of-tree
DEC: dispersal-extinction-cladogenesis
DIVA: dispersal-vicariance analysis
FP: Fitch parsimony
HIV: human immunodeficiency virus
MCMC: Markov chain Monte Carlo
ML: maximum likelihood
SM: stochastic mapping
UPGMA: unweighted pair group method with arithmetic mean
